# Suboptimal use of non-vitamin K antagonist oral anticoagulants: Results from the RAMSES study: Erratum

**DOI:** 10.1097/MD.0000000000006206

**Published:** 2017-02-17

In the article “Suboptimal use of non-vitamin K antagonist oral anticoagulants: Results from the RAMSES study”,^[1]^ which appeared in Volume 95, Issue 35 of *Medicine*, section 2.4 contained a bullet list which incorrectly listed 6 definitions. The first 3 of these definitions were listed incorrectly. The section should have read as follows:

The patients were categorized into 3 groups according to these criteria:

- AT: patients, those received recommended dose as per guideline,
- UT: patients, those were on the lower dose of NOAC than the recommended dose, and
- OT: patients, those were on the higher dose of NOAC than the recommended dose.

The parameters studied were compared among AT, UT, and OT groups.

In addition, the sentence “For AT, UT, and OT groups, the respective mean CHA_2_DS_2_VASc scores were significantly different 3.4 ± 1.4, 3.4 ± 1.3, and 4.2 ± 2.5 (*P* < 0.001) and the respective mean HASBLED scores were also significantly different 1.6 ± 1.0, 1.3 ± 0.6, and 2.5 ± 1.1 (*P* < 0.001), respectively” should have been deleted from section 3.1 of the article.
